# Stimuli-Responsive Gold Nanoparticles for Cancer Diagnosis and Therapy

**DOI:** 10.3390/jfb7030019

**Published:** 2016-07-21

**Authors:** Li Tian, Linfeng Lu, Yang Qiao, Saisree Ravi, Ferandre Salatan, Marites P. Melancon

**Affiliations:** 1 Department of Interventional Radiology, The University of Texas MD Anderson Cancer Center, 1515 Holcombe Boulevard, Houston, TX 77030, USA; LTian2@mdanderson.org (L.T.); john.yang.qiao@gmail.com (Y.Q.); ferandre@gmail.com (F.S.); 2 Department of Chemical and Biomolecular Engineering, Rice University, 6100 Main Street, Houston, TX 77005, USA; ll32@rice.edu; 3 Department of BioSciences, Rice University, 6100 Main Street, Houston, TX 77005, USA; sr56@rice.edu; 4 Graduate School for Biomedical Science, University of Texas Health Science Center at Houston, 6767 Bertner Ave., Houston, TX 77030, USA

**Keywords:** gold nanoparticle, cancer, tumor, stimuli-responsiveness, extracellular pH, redox, extracellular matrix

## Abstract

An emerging concept is that cancers strongly depend on both internal and external signals for growth and invasion. In this review, we will discuss pathological and physical changes in the tumor microenvironment and how these changes can be exploited to design gold nanoparticles for cancer diagnosis and therapy. These intrinsic changes include extracellular and intracellular pH, extracellular matrix enzymes, and glutathione concentration. External stimuli include the application of laser, ultrasound and X-ray. The biology behind these changes and the chemistry behind the responding mechanisms to these changes are reviewed. Examples of recent in vitro and in vivo studies are also presented, and the clinical implications of these findings are discussed.

## 1. Introduction

**Scheme 1:**
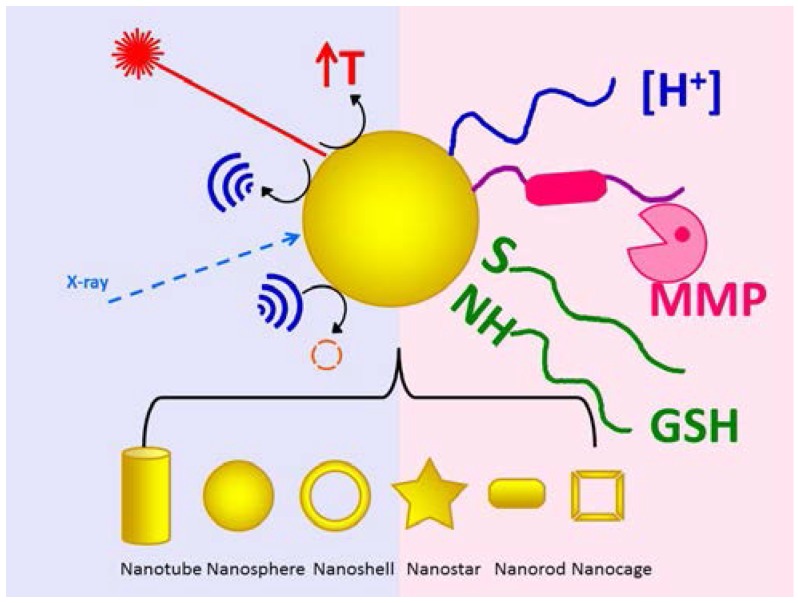
Review of gold nanoparticles (AuNPs) responsive to stimuli. On the left (blue region) are the reviewed external stimuli, including laser, ultrasound and X-ray, as well as photoacoustic imaging. On the right (pink region) are the reviewed intrinsic stimuli, including pH, matrix metalloproteinases (MMP), and redox condition (GSH).

Gold nanoparticles (AuNPs) have long been studied for their potential in facilitating anticancer therapy. Several extraordinary reviews on the synthesis of AuNPs [1], and their application in cancer diagnosis and therapy have been published [2,3]. Here, we review stimuli-responsive AuNPs that can be activated either intrinsically or extrinsically. The diversity in the design of AuNPs and their stimulus-responsiveness makes them promising multifunctional nanoplatforms. In this article, we review the most commonly used stimuli in cancer research and examine how AuNPs have been developed and studied as a multifunctional nanoplatform for cancer theranostics (Scheme 1). 

Intrinsic stimuli are micro-environmental differences that occur either pathologically or physiologically (Scheme 1). They include pH, extracellular matrix metalloproteinases (MMPs), and glutathione (GSH) that regulate the intracellular redox condition. Sensitivity to an individual stimulus is mainly caused by the incorporation or surface modification of polymers and peptides sensitive to the particular stimulus, such as pH-sensitive polymers, peptide sequences that can be cleaved specifically by MMPs, and disulfide bond-containing polymers. The introduction of a stimulus-sensitive component can increase the delivery of a chemotherapeutic agent or cause a spectrum change that can help to quantify molecular changes in tumors. This section covers the pathological and physiological causes of changes in the tumor microenvironment, the rationale and hypothesis underlying the design of each AuNP, and, briefly, the results of these studies and their implications for cancer diagnosis and treatment. A brief summary of the AuNPs that are responsive to intrinsic stimuli is presented in Table 1, Table 2 and Table 3. The majority of the work regarding intrinsic stimuli is still in the preclinical phase.

External stimuli include lasers and ultrasound, both of which are used clinically to treat cancer patients. AuNPs are also used in photoacoustic (PA) imaging. External stimuli activate certain physical properties of AuNPs, such as their surface plasmon resonance (SPR). The SPR can be fine-tuned by adjusting the shape and size of the AuNP, for example, the diameter of nanospheres, aspect ratio of nanorods, or thickness of nanoshells. Because the SPR is fine-tunable, AuNPs powerfully enhance hyperthermia when combined with lasers. Similarly, AuNPs can decrease the cavitation threshold of ultrasound. PA imaging, in which laser excitation is converted to ultrasound emission, is also discussed. Enhancement and sensitization of X-ray radiation by AuNP is also reviewed. 

## 2. Intrinsic Stimuli

### 2.1. pH

Tumors are slightly acidic because they are often hypoxic. Despite the release of angiogenesis factors that form neovasculature [4,5,6,7,8,9], tumor tissue often requires more oxygen and nutrition than can be supplied by the neovasculature. The oxygen concentrations in blood, healthy tissue, and typical hypoxic solid tumors are 10%–12.5%, 3%–6%, and 1%–2%, respectively [10,11]. Hypoxia triggers many alterations in gene expression and metabolism in cells, including the upregulation of hypoxia-inducible factor 1 [11,12], which leads to the overexpression of glycolytic enzymes and glucose transporters (GLUT1 and GLUT3) [13]. Glucose molecules are more actively internalized and converted to pyruvate as a result. Interestingly, in tumor cells, the majority of the resulting pyruvate undergoes a truncated pathway and is converted to lactic acid directly, instead of entering the tricarboxylic acid cycle. This phenomenon is known as the Warburg effect and has been observed in tumor cells even in normoxia [14,15]. Hypoxia also triggers the upregulation of Na+/H+ exchangers, which play an important role in maintaining intracellular pH [16,17], pumping large quantities of protons formed during glycolysis out of cells. These overproduced protons would normally be washed out by blood flow and lymphatic drainage, and the extracellular pH would remain normal [18]. However, because of the decreased blood flow [19] and impaired lymphatic drainage [20] in tumor tissues, the excess protons accumulate in the extracellular space [19]. Although the intracellular pH remains close to normal, at around 7.2, studies have shown that the extracellular pH in several kinds of tumors is more acidic, with an average pH of around 6.8 [21,22]. 

**Figure 1:**
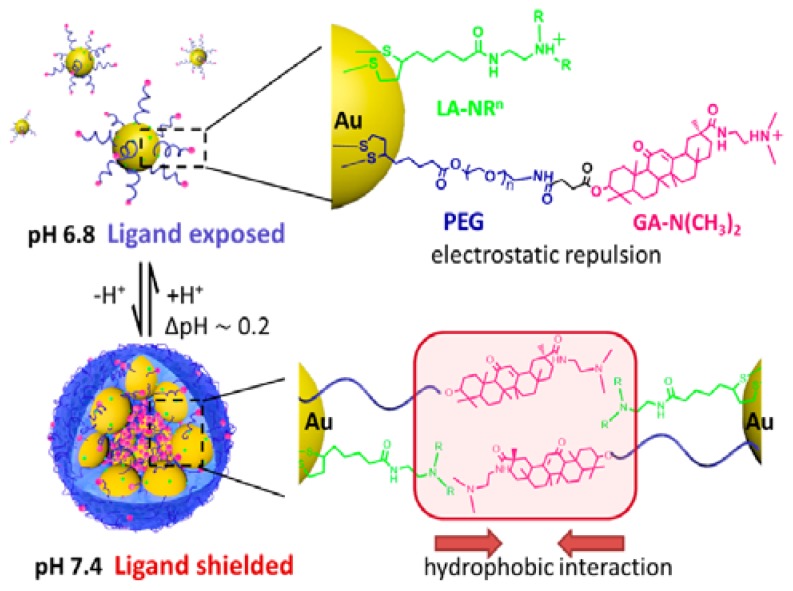
Schematic Illustration of GA Ligand’s Shield at Blood pH (pH 7.4) and Exposure at Tumor Extracellular Environment pH (pH 6.8), and Possible Mechanism of Sharp pH-Responsive Assembly and Disassembly of Au NPs. Reprinted with permission from [23]. Copyright American Chemical Society, 2014.

AuNPs are made sensitive to pH by using a polymer that has a different charge at different pH values, by adjusting the SPR, or by combining these two techniques (Table 1). For example, shieldable tumor-targeting AuNPs that possessed a self-assembly/disassembly property triggered by extrinsic pH changes were prepared by surface modification of AuNPs with two other components, lipoyl tertiary amines (LA-NRn) and poly(ethylene glycol) (PEG) modified by a glycyrrhetinic acid (GA) derivative, forming PEG-GA-N(CH_3_)_2_ [23]. GA is a targeting ligand of hepatocytes. It also serves as the tuner of hydrophobicity in these AuNPs. The carboxylic acid group on GA was modified into a tertiary amine group, causing the modified GA moiety to transit from non-charged (hydrophobic) to positively charged (hydrophilic) as pH dropped, and LA-NRn underwent a similar transition (illustrated in Figure 1). This hydrophobic/hydrophilic transition resulted in the aggregation of the AuNPs and the hiding of the targeting ligand at pH 7.4, and disassembly and exposure of the targeting ligand at acidic pH (such as 6.8). The results of an in vitro study showed that the uptake of AuNPs@LA-NR4+PEG-GA-N(CH_3_)_2_ by HepG2 cells was about three times higher at pH 6.8 than at pH 7.4. Aside from the incorporation of a polymer/compound that has a different charge at different pH values, chemical bound that is prone to hydrolysis has also been used to introduce pH sensitivity. The hydrolysis of the citraconic amide moiety triggers the release of doxorubicin (Dox) to the tumor area and spurs the aggregation of AuNPs for photothermal therapy [24]. 

Like extracellular pH, the average intracellular pH is slightly lower than the physiological pH of 7.4. Moreover, the pH value differs among subcellular compartments [25,26]. Some compartments, in particular endosomes and lysosomes, even possess a pH as low as 4 or 5. The lower intracellular pH plus the different extracellular and intracellular ion concentrations have provided a theranostic target. For example, Cha et al. [27] fabricated theranostic hybrid nanoparticles from AuNPs, methoxy-poly(ethylene glycol) (mPEG)-Asp-Cys copolymers, and calcium phosphate (CaP) [27]. The employed biological niches were the lower lysosomal pH (pH 4.5) versus physiological pH (pH 7.4), the decreased intracellular calcium ion concentration (reduction of 104 times compared to extracellular conditions), and the increased phosphate ion concentration (increase of 40–70 times compared to extracellular conditions) [28]. The hypothesis was that the lower pH and the decreased calcium concentration would facilitate the dissolution of the calcium layer and therefore increase the release of Dox in lysosomes. In the in vitro test, the particles stayed stable at physiological pH (7.4) and released only 13% of Dox after 12 hours, but 70% of the Dox at pH 4.5. In vitro work also showed an increased cytotoxicity on HeLa cells. The authors proposed future in vivo work on the theranostic effect of PEGylated Dox-AuNP@CaP particles. Computed tomography (CT) imaging could be obtained and Dox delivered using AuNPs.

### 2.2. Matrix Metalloproteinases

MMPs are zinc-dependent proteins that belong to the metzincin superfamily [29]. Twenty-four MMPs have been identified [30]. They are synthesized as pre-proenzymes in cells, and most are secreted into the extracellular space as proenzymes [31]. MMPs used to be viewed as “scissors” that break down the basement membrane, degrade extracellular matrix components, and facilitate tumor invasion and metastasis [32,33]. Recent research, however, has revealed that MMPs are involved in several different aspects of the regulation of the tumor microenvironment [34]. Besides participating in tumor invasion and metastasis, MMPs are also involved in cell growth, angiogenesis, and apoptosis. They can be produced by both tumor cells and surrounding stromal cells [35,36]. MMP inhibitors have attracted growing interest in clinical trials [36], and various cleavage sequences of MMPs have been incorporated into the design of new anticancer nanoparticles, including AuNPs. Cleavage of these sequences often causes an optical change in the AuNPs, and this change is, in turn, mathematically related to the amount and/or activity of the MMPs (Table 2).

One application of AuNPs is the bench quantification of MMP. For example, Kim et al. performed a colorimetric assay of MMP activity using AuNPs [37]. Based on the discovery of a high binding affinity of His-tags with densely packed carboxyl groups on the nanoparticle surface in the presence of metal ions, they hypothesized that the carboxyl AuNPs would self-assemble in the presence of both metal ions and peptides with hexahistidine at both ends (H6-pep-H6). This self-assembly would cause a blueshift in the solution’s spectrum. When the peptide sequence in H6-pep-H6 was designed to be the cleavage sequence of MMP, MMP would cleave the peptide and reverse the self-assembly and the spectrum shift. The color extinction ratio of E520/E700 was calculated and used to characterize MMP activity. 

Localized SPR has also been studied to detect the activity of membrane type 1 MMP (MT1-MMP) [38]. Cleavage of MT1-MMP specific peptide sequence caused a blueshift of the maximum wavelength of localized SPR; this shift was also greater at a higher concentration of MT1-MMP. However, the shift could not be quantified as a function of MT1-MMP concentration.

Similarly, an MMP assay using biofunctionalized AuNPs and a nitrilotriacetic acid-modified chip has also been developed [39]. AuNPs were functionalized by a 15-peptide sequence, which served as the sensing element and contained an MMP-7 substrate sequence and a six-histidine (His) tag. With the presence of Ni2+, the resulting peptide-AuNPs could be trapped on the nitrilotriacetic acid chip on the His end. MMP-7 could proteolyse the MMP-7 substrate sequence and cleave AuNPs from the chip. After silver enhancement, the amount of remaining bound AuNPs could be converted into grayscale for quantification. The scanometric readout could then be used to quantify MMP-7 concentration. The authors proposed that the assay could also be used to detect a wider range of MMPs if other MMP-specific sequences were used. The advantage of this assay is that it could be performed in full cell culture medium. 

Besides quantification of MMP concentration or activity, AuNPs have also been fuctionalized to enhance cellular uptake. For example, MMP2-functionalized AuNPs have been engineered [40]. PEG was linked with AuNPs via a peptide sequence, PVGLIGC, which was cleavable by MMP-2 (cleavable AuNPs). After incubation with MMP-2, cleavable AuNPs aggregated, and the average hydrodynamic diameter increased from 26 nm to 311 nm, with a drop of the average zeta potential from −9 to −45 mV and a redshift in the spectrum. Cellular uptake of cleavable AuNPs also increased with increasing concentrations of MMP-2. The authors also suggested that the liver uptake of PEGylated nanoparticles could be a disadvantage, but they did not address this issue in the paper. It will also be interesting to see what happens when chemotherapeutic agents are incorporated into the system in an in vivo study. 

**Figure 2:**
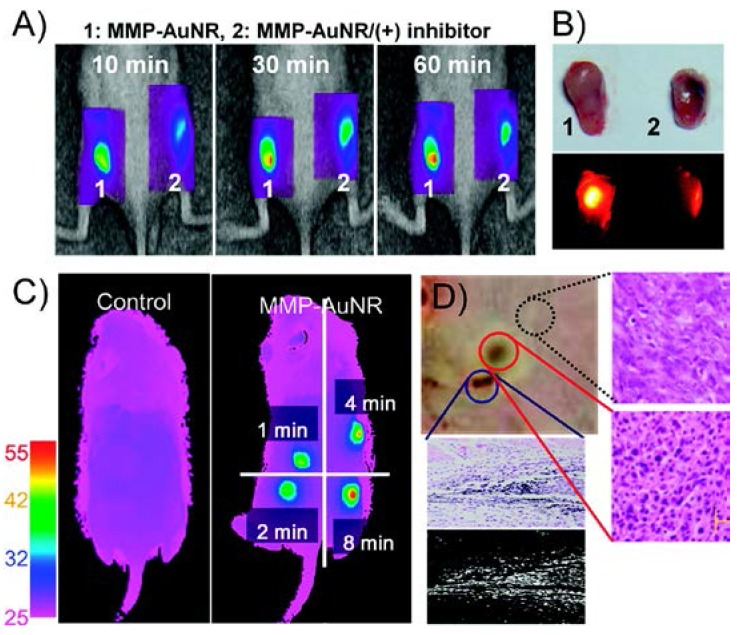
(**A**) NIRF tomographic images of SCC-7 tumor-bearing mice after intratumoral injection of the MMP-AuNR probe without (1) and with (2) inhibitor; (**B**) Optical and NIRF images of excised tumor after injection of MMP-AuNR without and with MMP-2 inhibitor; (**C**) Infrared thermal images of tumor-bearing mice according to different laser irradiation times. AuNR was injected intratumorally prior to laser irradiation; (**D**) Optical images of exterior of SCC-7 tumor irradiated by laser. Histology (right) and dark field image (lower) of tumor after treatment are also presented. Reprinted with permission from [41]. Copyright American Chemical Society, 2010.

The intrinsic properties of AuNPs have also been incorporated into the design to facilitate ablation. For example, MMP-sensitive gold nanorods (MMP-AuNRs) were designed for simultaneous imaging and photothermal ablation for cancer [41]. The peptide sequence GPLGVRGC was used to link a near-infrared (NIR) fluorescent (NIRF) dye, Cy5.5, onto the surface of AuNRs. This peptide sequence could be degraded by MMP. In the resulting MMP-AuNRs, the NIRF was quenched until the peptide was cleaved by MMP and the Cy5.5 was released. To avoid tissue absorption, the AuNRs’ aspect ratio was adjusted so that the strong absorption was tuned to the NIR region. Conjugation with the Cy5.5 peptide sequence did not change the stability or photothermal properties of the MMP-AuNRs. Incubation of MMP-AuNRs with MMP-3, -7, -9, and -13 resulted in increased fluorescence, though MMP-7 treatment did not increase the fluorescence as much as the rest of the MMPs. After irradiation with a continuous-wave laser at 671 nm for 10 minutes, MMP-AuNR-treated cells underwent cell damage and apoptosis. The in vivo efficacy of MMP-AuNRs was also demonstrated on SCC-7 tumor xenografts on nude mice. Enhanced NIRF was observed after intratumoral injection of MMP-AuNRs. Maximum NIRF was observed 60 minutes after the injection. Pre-injection of an MMP inhibitor reduced the NIRF. Hyperthermal therapy was also administered, with tumor temperature increasing by more than 45 °C after 4 minutes of treatment. More tumor cell damage was observed in the group of mice co-treated with MMP-AuNRs than in the group treated with hyperthermal therapy alone (Figure 2). 

Other research has incorporated more than one targeting mechanism. For example, dual-functionalized AuNPs that are responsive to both MMP-2 and intracellular GSH have been developed [42]. In this study, Dox was attached to a peptide sequence CPLGLAGG, which was cleavable to MMP-2. This peptide sequence was linked to the surface of AuNPs via a thiol-Au bond. The surface of the AuNPs was also decorated with PEG for improved stability and prolonged blood circulation. The authors hypothesized that once these AuNPs (called Dox-substrate/AuNP) reached the tumor site, some Dox would be released due to the cleavage of the peptide sequence. They expected to observe increased fluorescence because Dox would change from a quenched to an excited state. They also anticipated cytotoxicity from the released Dox. The remaining Dox would be released into the cytosol after the AuNPs were internalized, due to the thiol exchange between cytosolic GSH and the thiol-Au bond. The intensity of cleaved peaks on high-performance liquid chromatography gradually increased over 40 hours. Fluorescence emission spectra of Dox-substrate/AuNP also revealed a gradual increase in Dox emission intensity over a period of 84 hours during which Dox-substrate/AuNPs were incubated with MMP-2. Similarly, incubation of Dox-substrate/AuNP with GSH also revealed a gradual but steady increase of the fluorescence intensity of released Dox over 72 hours. After 100 hours of incubation, MMP-2 released around 80% of Dox and GSH around 60%. In both SCC-7 and HT-29 cell lines, Dox-substrate/AuNP was endocytosed by the cells and exhibited cytotoxicity. The efficacy of Dox-substrate/AuNP was further evaluated on nude mice bearing SCC-7 tumors. Dox fluorescence could be observed in tumor regions 30 minutes after Dox-substrate/AuNP was subcutaneously injected. The fluorescence of Dox faded significantly if the animals were pre-injected with an MMP-2 inhibitor, TIMP-2. The antitumor activity evaluation suggested that Dox-substrate/AuNP had similar antitumor efficacy to, but lower toxicity than, free Dox. This research took advantage of not only MMP expression in the tumor region, but also the higher cytosol GSH concentration, which we will discuss in the following section.

### 2.3. Glutathione

GSH has a tripeptide structure, with a free thiol group on the side of the molecule. It is the predominant intracellular nonprotein sulfhydryl in animals [43]. GSH exists in higher concentrations in the cytoplasm and the nucleus (0.5–20 mM) [44,45,46] than in in the extracellular fluid (2–20 μM) [47,48]. The importance of GSH lies in its thiol-thiol exchange property, which can break down disulfide bonds and substitute ligands attached on Au surfaces via thiol-Au or other bonds (Table 3).

The most commonly used GSH trigger mechanism is the thiol-thiol exchange between the thiol of GSH and the thiol-Au bond. At intracellular GSH concentrations, the ligand that was once attached on the Au surface via a ligand-thiol-Au bond is cleaved by the substitution of a GSH-thiol-Au bond. For example, per-6-thio-β-cyclodextrin (CD) was linked to the surface of AuNPs, which were further stabilized by PEG [49]. GSH-mediated release was characterized by using rhodamine B-conjugated SH-CD (RhoCD) on MCF-7 and A549 cell lines. A549 cells had a higher cytosol GSH level, and a higher fluorescence from restored rhodamine B was observed. A549 cells also had higher epidermal growth factor (EGF) receptor expression. Thus, when the AuNPs were also functionalized by EGF, increased fluorescence was observed on A549 cells. When β-lapachone was carried in the hydrophobic pockets of SH-CD, AuNP/lap both with and without EGF induced apoptosis in A549 cells, and AuNP/lap with EGF induced a higher percentage of apoptosis. Similarly, the surface of AuNPs was functionalized via thiol (sulfhydryl) bond with CD, rhodamine B-CD, methoxy-poly(ethylene glycol sulfhydryl) (mPEG-sulfhydryl), and/or heterofunctionalized thiol-PEG-Biotin [50]. The hydrophobic pockets of CD were used to incorporate hydrophobic drugs such as paclitaxel (PTX). GSH facilitated the release of PTX, which in turn induced cytotoxicity. The cytosol fluorescence intensity of cells treated with AuNPs modified with rhodamine B-CD was also restored.

Similarly, a thiolated dye (HSBDP) and a tetra(ethylene glycol)-lated cationic ligand (TTMA) were attached to the surface of AuNPs [51]. HSBDP was used as an analogue of a hydrophobic drug. The fluorescence of HSBDP was quenched when it was attached to the surface of AuNPs but was increased due of the release of HSBDP by GSH. TTMA was designed to enhance cellular uptake. Hep G2 cells were incubated with AuNPs for 4 h, and strong fluorescence from HSBDP was observed after 96 hours. The cytosol fluorescence could also be increased by adding a GSH monoester (GSH-Oet), which could be internalized and hydrolysed into GSH. The release of the dye was also specifically triggered by GSH, as removal of the thiol group from GSH did not increase the fluorescence of the incubated solution.

Other polymeric architectures have also been used to modify AuNPs. For example, dendrimer-encapsulated gold nanoparticles (DEGNPs) have been used as drug carriers [52]. Both anticancer drugs that have intrinsic thiol groups (thiol-containing drugs) and drugs from which thiol groups are extended (thiolated drugs) can be incorporated in DEGNPs via the thiol-Au bond. DEGNPs were shown to be more stable than AuNPs because the Au surface was protected by the dendrimer.

In another study, [53], polysaccharide heparin was functionalized with thiol groups and grafted with pheophorbide A (PhA), a photosensitizer. The resulting PhA-conjugated heparin (PhA-H) was used to coat AuNPs via a thiol-Au bond, and PhA-H/AuNPs were obtained. When PhA-H/AuNPs were treated with 10 mM of GSH, the once-quenched fluorescence of PhA was recovered. In addition, restored were PhA’s photoactivity and generation of singlet oxygen upon laser irradiation. Phototoxicity was examined on A549 cells. PhA-H/AuNP-treated cells had a higher fluorescence signal than free PhA-treated cells. PhA-H/AuNPs also induced higher rates of cytotoxicity than free PhA. In vivo efficacy was examined using a murine xenograft model of A549 tumors. The fluorescence signal was observed from 1 to 72 h after intravenous injection in both PhA-H/AuNP-treated and free PhA-treated animals. The fluorescence signal from free PhA was distributed throughout the animal, while the PhA-H/AuNP signal was mainly localized at tumor sites. Tumor volume and weight were also lower in PhA-H/AuNP-treated mice than in free PhA-treated mice and untreated control mice.

**Figure 3:**
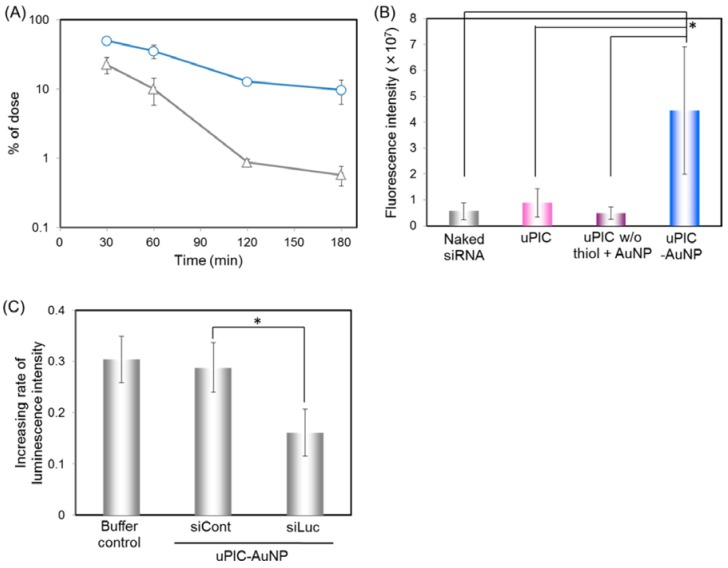
(**A**) Blood circulation property of uPIC-AuNPs (open circle) and bare AuNPs as a control (open triangle) determined by ICP-MS. Results are expressed as mean and standard deviation (*n* = 3–4); (**B**) Subcutaneous HeLa-Luc tumor accumulation of Alexa-siRNA delivered by each formulation at 4 h after intravenous injection (4.8 μg siRNA/mouse), determined by IVIS. Results are expressed as mean and standard deviation (*n* = 4, * *p* < 0.01); (**C**) Increasing rate of luminescence intensity (IR_LI_) from subcutaneous HeLa-Luc tumors after treatment with siLuc- or siCont-loaded uPIC-AuNPs (5.8 μg siRNA/mouse/shot) or a Hepes buffer control. The IR_LI_ values were calculated as an indicator of luciferase gene silencing activity, as described in the Materials and Methods. Results are expressed as mean and standard error of the mean (*n* = 4, * *p* < 0.05). Reprinted with permission from [54]. Copyright American Chemical Society, 2014.

GSH-responsive AuNPs have also been studied for their potential use in the delivery of large molecules. For example, short interfering RNAs (siRNAs) were combined with a poly(ethylene glycol)-b-poly(L-lysine)-thiol (PEG-PLL-SH) copolymer to form a single siRNA-loaded unimer polyion complex (uPIC) [54]. This uPIC was then linked onto the surface of AuNP by a thiol-Au bond. Electrophoresis results suggested that GSH enhanced the release of siRNA from the uPIC-AuNPs. In vivo results showed a higher silencing percentage of luciferase in HeLa-Luc cells treated with uPIC-AuNP than in those treated with uPIC alone. In vivo efficacy was studied by intravenously injecting the particles into the tail vein of mice bearing subcutaneous HeLa-Luc tumors. Four hours after injection, the accumulation of siRNA in the tumor site was the highest in the uPIC-AuNP group (Figure 3).

GSH is often used to exchange the thiol-to-thiol ligand to release drug molecules incorporated on AuNPs because thiol-containing molecules are typically used to passivate the surface of AuNPs. However, the ligand exchange has been extended to other functional groups, providing that they have weaker affinity to Au than thiol. For example, Au has been passivated with amine-containing anticancer drug molecules, and GSH facilitated the release of the drugs via thiol-to-amine exchange [55]. The authors tried quite a few amine-containing anticancer drugs to passivate the AuNPs and found a molecular weight of 300 to be the threshold for the successful formation of colloidally dispersed AuNPs. Methotrexate (MTX) was cited as an example in the article. The Au:MTX particles were found to be stable at pH 4–9. When Au:MTX was incubated with GSH, MTX was released via thiol-to-amine exchange. Although the study did not fully address the physiological relevance of the tested GSH concentrations, the antitumor effect of Au:MTX was demonstrated both in vitro and in vivo.

Besides amine-Au bonds, purine and pyrimidine compounds can also be absorbed onto Au surfaces via nitrogen groups, and this absorption can also be exchanged by cytosol GSH. In one study, 6-thioguanine (6TG), gemcitabine, acycloguanosine, and fadrozole were used to coat AuNPs. Gemcitabine, acycloguanosine, and fadrozole interacted with AuNPs via their purine or pyrimidine groups, and 6TG interacted with AuNPs via both sulfur and nitrogen atoms [56]. Surface-enhanced Raman scattering spectra showed decreased peak intensities of all the drug-containing AuNPs after GSH treatment, which indicated dissociation and release of the drug molecules. However, over 30 minutes of treatment with 2 mM of GSH, the spectral intensities of 6TG-AuNPs did not decrease as substantially as those of the rest of the drug-containing AuNPs. This effect was partially attributed to the stronger association of 6TG with Au surface via its sulfur atom. Gemcitabine, acycloguanosine, and fadrozole appeared to have been detached from the AuNPs and released into the cytosol, whereas the majority of 6TG-AuNPs remained undetached in the endosomes or lysosomes.

Besides their major role as drug carriers, AuNPs could also be used in an auxiliary role. For example, AuNPs have been used to assist in stabilizing Pluronic micelles (PF-micelles) [57]. PTX was loaded in the hydrophobic core of the PF-micelles to form PF-PTX-micelles. The hydrophilic outer shell layer was thiolated and crosslinked by neighboring AuNPs via the Au-thiol bond, producing AuNP-crosslinked PF-PTX-micelles (Au-PF-PTX-micelles). This crosslinking increased the stability of the PF-micelles. PTX release from Au-PF-PTX-micelles was accelerated by GSH, and PF-PTX-micelles were more cytotoxic than were Au-PF-PTX-micelles to human glioma cell line U87. This higher cytotoxicity was explained as inhibition of release of PTX owing to the crosslinking in Au-PF-PTX-micelles. Results from systemic administration of the two micelles also showed that Au-PF-PTX-micelles had a longer systemic circulation time and were cleared from plasma more slowly than PF-PTX-micelles. PTX accumulation was high in the spleen and liver and low in the kidneys and lungs after systemic administration of Au-PF-PTX-micelles.

## 3. External Stimuli

### 3.1. Laser

Laser light is introduced into a patient through optical fibers fitted in an endoscope; this treatment is commonly used to kill superficial cancers. The direct cause of cell death is hyperthermia generated by the light beam. Laser therapy is nonselective and loses energy as the beam penetrates deeper tissues.

**Figure 4:**
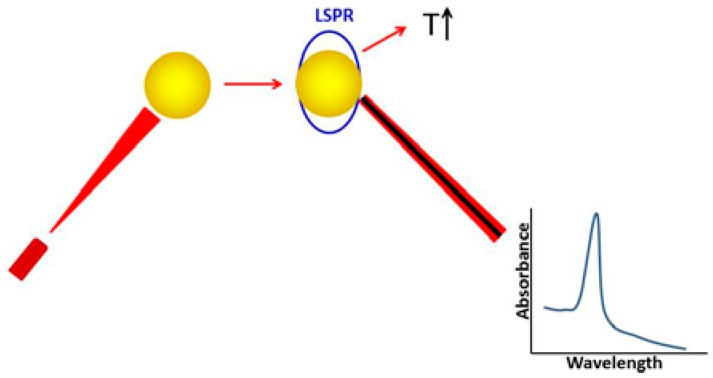
Illustration of localized surface plasmon resonance (LSPR) of AuNPs.

AuNPs exhibit a rapid photothermal conversion owing to their SPR (Figure 4). This photothermal conversion can be used to heat a localized area surrounding the AuNPs by administering light at a frequency that overlaps with the AuNPs’ SPR absorption. The SPR properties of noble metal nanoparticles, including AuNPs, and their applications to biosystems have been reviewed elsewhere [58]. AuNPs have also been undergoing intense study of their potential use in combination with laser for theranostic purposes. Research has focused on fine-tuning the photothermal properties of AuNPs by controlling their structure, shape, size, tumor specificity through ligand conjugation, combinations of other nanostructures and platforms, and so on. Many excellent reviews on this topic have been published [59,60,61,62,63,64,65,66]. Here we provide a review of recent progress (Table 4).

Among the most studied AuNPs are nanospheres, nanorods [67], nanoshells [68], and nanostars [69]. Shape and size play an important role in determining the photothermal profile of AuNPs. In one study, Au nanostars of 25–150 nm were synthesized [69]. The SPR peak shifted from 500 to 1000 nm as the size of the nanostars increased. The photothermal heating efficiency also varied according to the nanostar size and the wavelength of the laser source. However, the photothermal heating efficiency was found to be less size- and wavelength-dependent once the nanostars were internalized into the cells. This attenuation was explained as nanostar aggregation as the particles were confined in the endosome upon internalization. Similarly, attenuation was also found after cellular internalization on animal xenografts. The authors inferred that the size and coating of the nanoparticle were more important than the experimentally measured SPR peak, as the proper size and coating could ensure optimal biodistribution.

Branched AuNPs were also synthesized by the supramolecular aggregates of deoxycholate bile acid in Au solution. The morphology and the maximum absorbance peak of the branched AuNPs were dependent on the deoxycholate bile acid concentration, and gelation was observed at higher acid concentration. Conjugation of cyclic RGD (cRGD) enhanced cellular uptake and, when combined with NIR irradiation, increased in vivo antitumor efficacy [70]. AuNPs were grown on carbon nanotubes and increased cytotoxicity in combination with NIR irradiation [71]. 

Nanorods and nanocages have also been compared. In one study, PEGylated nanorods of 60 × 14.8 ± 6.5 × 2.0 nm and PEGylated nanocages of 50 ± 7 nm were synthesized [72]. Nanocages exhibited a higher light-to-heat transduction efficiency and required 18.4 times fewer particles (approximately half the Au mass) than nanorods to achieve the same heating profile. Nanocages also had higher cell internalization than nanorods, but the internalized amount was dependent on cell type. Biodistribution in xenograft prostate tumor-bearing mice suggested that nanocages and nanorods had different uptake and residence time in blood, heart, kidneys, liver, lungs, spleen ant tumor; long-term biodistribution in healthy animals suggested that nanocages had a higher excretion rate.

Surface modification has also been studied. For example, modification with the TAT peptide was found to increase the intracellular delivery of Au nanostars and consequently increase the efficiency of photothermolysis [73]. Conjugation of Au nanostars with both cRGD and Dox not only enabled the delivery of Au-cRGD-Dox close to the nucleus and facilitated its entrance into the nucleus, but also increased antitumor efficacy after irradiation with NIR light [74]. Other surface modifications with macromolecules include conjugation with anti-Mucin 7 antibodies [75], albumin [76], and others. Small molecules have also been used for surface modification. For example, Prussian blue has been used to coat AuNPs to achieve photothermal ablation and simultaneous PA/CT bimodal imaging [77].

AuNPs have also been combined with other nanostructures and treatment modalities to achieve multifunctionality and synergistic effect in cancer theranostics. For example, hydrophobic AuNPs of 3–7 nm have been incorporated into the lipid bilayer of liposomes to fabricate photothermally responsive liposomes [78]. The in vitro-triggered fluorescein release from these liposomes was five times higher than the fluorescein release from non-responsive liposomes. The fluorescein retention in the tumor was higher in the responsive liposome-treated group (81%) than in the free fluorescein-treated group (14%) after 2 h. AuNP-coated liposomes and their pharmacokinetic profiles have also been studied [79].

A quadrapeutics system, in which AuNPs were combined with other nanoparticles and treatment modalities, has also been also studied [80]. EGFR-conjugated AuNPs and therapeutic nanocarriers were administrated systemically, and the AuNPs and therapeutic nanocarriers were aggregated intracellularly into a mixed cluster after endocytosis. Then a single laser pulse was administered to generate plasmonic nanobubbles, which caused cell death and also released therapeutics from the nanocarriers. A single X-ray dose was then administered and absorbed by AuNP nanoclusters. Synergistic amplification of the X-ray was observed when a chemotherapeutic agent was carried by the nanoparticles. This quadrapeutics system was found to be more effective than chemoradiation in treating highly resistant and aggressive tumors. This greatly increased effectiveness was explained as the combination result of the mutual enhancement of chemotherapy and radiation therapy and of mechanical radio- and chemosensitization. Cancer specificity was also achieved from ligand targeting.

Graphene oxide (GO) and its derivatives have also been incorporated with AuNPs. Reduced GO (rGO) possesses high photon-thermal transfer efficiency upon NIR irradiation. It has been incorporated into a hydrogel system consisting of rGO, amaranth extract, and AuNPs [81]. The hydrogel was formed by the reduction of GO to rGO by amaranth extract, then NIR irradiation of rGO and AuNPs. Amaranth extract served multiple roles, including photosensitization, while rGO and AuNPs further improved 1O2 generation. The cytotoxicity of this system against cancer cells was demonstrated in vitro. In a separate study, microRNA-122-loaded AuNPs on graphene nanocomposites were modified with P-glycoprotein antibodies and folic acid [82]. This system inhibited tumor growth in a xenograft model. Similarly, anti-EGFR-conjugated nanosheets containing rGO/mesoporous silica/AuNPs also increased the efficacy of photothermal therapy by enhancing the synergistic effect of conjugated AuNPs and rGO nanosheets [83].

Cell technologies have also been used to enhance the accumulation and intratumoral distribution of AuNPs. For example, human induced pluripotent stem cells (iPS) with C-X-C chemokine receptor type 4 (CXCR4) have been used to load Au nanorods@SiO_2_@CXCR4 to achieve better tumor target migration, with CXCR4 used to increase loading [84]. This AuNP-iPS platform preserved the photothermal properties of the Au nanorods and inhibited the growth of human gastric cancer MGC-803 tumors in a xenograft model. In another study, adipose-derived mesenchymal cells were loaded with superparamagnetic iron oxide coated with AuNPs (SPIO@AuNPs) [85]. The loaded mesenchymal cells were used to deliver SPIO@AuNPs for both the theranosis of liver injury and the photothermal ablation of hepatocellular carcinoma. In both cases, the loaded nanoparticles were nontoxic to the stem cells and maintained their photothermal properties.

Laser therapy also demonstrated unique effects on AuNPs compared to other ablative therapies. One article compared the intratumoral nanoparticle (Dox loaded nanoshells) uptake after nanoembolization among several different ablative modalities, including radiofrequency, electroporation, and laser-induced thermal therapy [68]. Structural deformation of the nanoshells was found only in the region ablated with laser-induced thermal therapy. This finding contributed to the understanding of how drugs are released from nanoshells when combined with laser-induced thermal therapy.

### 3.2. Ultrasound

Ultrasound has long been a powerful tool for various clinical applications, ranging from prenatal imaging [86] to cancer detection [87]. Ultrasound machines detect the reflection and refraction of sound waves through various media [88]. As an ultrasonic wave propagates through different types of materials, different variations of acoustic impedance are created [88]. The differences in the reflection and refraction of the sound waves are then fed through an algorithm to paint a picture of the relative densities and shapes of the various tissues [88]. Diagnostic ultrasound is generally within the frequency range of 2–20 MHz and therapeutic ultrasound between 0.5 and 2 MHz [89,90]. A summary of AuNPs that are responsive to ultrasound is in Table 5. 

One ultrasound modality of great research interest is high-intensity focused ultrasound (HIFU). The intensity of HIFU is several orders of magnitude greater than that of standard ultrasound. A review of the various uses of HIFU and therapeutic ultrasound has been published by Phillips et al. [91]. HIFU provides noninvasive treatment for tumors deep in the body [89,92,93]. A detailed discussion of the role of HIFU-mediated hyperthermia in the enhanced delivery of nanoparticles to tumors has been published by Frazier et al. [94]. In general, an ultrasonic beam is directed toward the target tissue from single-element transducers or phased arrays [89,92]. The beam can be focused in a small area toward a specific target [89]. The target tissue absorbs acoustic energy and increases in temperature, which may cause acoustic cavitation or radiation, which in turn may lead to cell necrosis or apoptosis [89,90,92,93,95]. If HIFU is applied with correct timing, inertial cavitation, or the rapid collapse of the vapor bubble, can ensue [95]. However, this therapy carries a risk of complications, such as adjacent tissue damage, skin burns, and organ system-specific side effects [89,93]. Furthermore, the development of bone and gas pockets can increase damage to surrounding tissue and cause difficulty in targeting specific areas [89,93]. The efficacy of HIFU can be improved by using it in conjunction with chemotherapy or radiotherapy [92].

Contrast agents or enhancement agents are often used to improve the efficacy of HIFU [93]. In the past decade, nanoparticles that serve both chemotherapeutic and imaging purposes have been developed [87]. AuNPs are used because they are biocompatible, easy to prepare, relatively nontoxic, and have a surface plasmon absorption property that make them good contrast agents [96]. They are particularly promising for use in altering the surface tension of microbubbles, increasing acoustic impedance, enhancing detectable scattering [93], and decreasing the threshold of cavitation intensity [97]. Cavitation caused by laser-heated nanoparticles has been shown to produce localized cell death. However, the laser fluence required to produce cavitation exceeds permissible levels or can cause permanent bleaching. Methods to lower the required fluence levels include aggregation of nanoparticles to cause thermal field overlap and plasmonic coupling and decrease of local pressure using an ultrasound field [98].

One study constructed AuNP-coated, polydopamine-modified poly(lactic-co-glycolic acid) (AuNPs@PDA/PLGA) hybrid capsules of an average size of 725 nm for use in ultrasound imaging and HIFU therapy [93]. The utility of these capsules for HIFU contrast imaging in vitro was tested in degassed water and ex vivo in bovine livers. The AuNPs@PDA/PLGA capsules rose more quickly in temperature when exposed to an ultrasonic probe compared to PLGA nanocapsules in pure water. The test particles reached 26 °C in 300 s. In vitro cytotoxicity test results showed that the AuNPs@PDA/PLGA capsules had no effect on cell mortality, which was comparable to that of cells placed in PBS. The echogenicity at a mechanical index of 0.6 under harmonic mode and 0.18 under contrast mode increased with the concentration of the nanoparticles, proving that the particles do act as contrast agents in ultrasonography. HIFU ablation with AuNPs@PDA/PLGA hybrid capsules showed an approximately four-fold or higher increase in damaged tissue compared to PLGA nanocapsules alone or a blank control of PBS only. This result is believed to be the effect of the increased thermal efficiency and conductivity of the AuNPs and the resultant mechanical and thermal effects of ultrasound.

The dependence of cavitation activity on HIFU pressure and laser energy was also studied using 82-nm AuNPs embedded in 7% acrylamide gel and tested with a 532-nm pulsed laser and 1.1-MHz transducer [95]. The results showed that laser irradiation on AuNPs lowered the HIFU cavitation pressure to as low as 0.92 MPa, compared to 4.50 MPa without the presence of the laser. When vapor bubbles were produced during the peak rarefaction phase, the cavitation activity was the highest. Furthermore, the threshold pressure decreased as the laser energy increased because the vapor bubble sizes became larger.

Another study showed that AuNP-coated, perfluorohexane-encapsulated and PEGylated mesoporous silica nanocapsule-based enhancement agents (MAPP) could be used for enhanced ultrasound imaging and HIFU enhancement [99]. AuNP-coated mesoporous silica nanocapsules were synthesized by S-Au chemistry and were then loaded with pyrene and perfluorohexane. Enhanced drug release was indicated by blue fluorescence in the cytoplasm of L929 murine fibroblast cells, showing that pyrene had been released from MAPP and into the interior of the cells. Cytotoxicity was shown to have increased by 20% in L929 cells after exposure to ultrasound; this was thought to have been caused primarily by heat effects, as the temperature of MAPP reached 54.8 ° in 2 minutes. Under both contrast and harmonic imaging modes, significant differences in the average grayscales between MAPP and PBS control and between pre- and post-MAPP injection were observed, attesting to the contrast-intensified ultrasound imaging provided by MAPP. Furthermore, injections of MAPP into hepatocellular carcinoma tumor-bearing nude mice showed that the nanoparticles accumulated in the tumors owing to enhanced permeability and retention effects. Finally, the presence of MAPP increased necrotic cell volumes significantly after treatment with HIFU ablation.

Combinations of multiple treatment modalities have also been studied [100]. An additive effect on epithelial breast cancer cell viability was shown when gold nanorods with a 4:1 aspect ratio, conjugated with mPEG and with a surface resonance peak at 813 nm was combined with laser therapy at 810 nm, ultrasound (50 kHz and 0.6/1/0 MPa), and Definity microbubbles. The AuNP and laser therapy mainly caused heat damage to cells that eventually led to necrosis, while ultrasound microbubbles created transient membrane pores that led to cell death.

Another ultrasound-based approach that has been adopted in cancer treatment is sonodynamic therapy [90,101]. Sonosensitizers like protoporphyrin IX are activated by ultrasound to cause inertial cavitation, which produces microscopic air bubbles within tissues [90,102]. The rapid collapse of the bubbles can cause excitation of the sonosensitizers to produce reactive oxygen species like superoxide anion, hydrogen peroxide, or hydroxyl radicals [90]. These species interact negatively within cells to cause dysfunction and, ultimately, apoptosis [90]. The use of AuNPs has shown to be a safe and efficient method to deliver the sensitizers to the target tissue and a means to provide a nucleation site for cavitation bubbles, decreasing the threshold intensity necessary for cavitation [102]. Furthermore, the nonradiative relaxation time of protoporphyrin is increased in the presence of AuNPs, a phenomenon that is favorable for the production of singlet oxygen, used for tissue destruction [102].

In one study, colon carcinoma tumors in BALB/c mice were used to test the efficacy of ultrasound and AuNP (7 nm)-protoporphyrin IX conjugates linked by a bidentate linker in producing cavitation and sonodynamic antitumor effects [102]. The researchers used cold degassed water as the ultrasonic medium in order to negate possible heat effects. The results showed significant antitumor effects, evidenced by reduced tumor volumes, increased survival rates of the mice, and greater amounts of necrotic tumor cells in the group of mice treated with ultrasound and the AuNP-protoporphyrin IX conjugate than in the groups that received various other combinations of ultrasound, protoporphyrin IX, and AuNPs. These results also showed that the conjugate is an effective sonosensitizer in sonodynamic therapy since there was negligible temperature increase; the antitumor effects were attributable to shear stress and cavitation during ultrasonic exposure.

Ultrasound has also been utilized in drug delivery. Ultrasound and microbubbles increase membrane permeability through sonoporation [100]. The microbubbles undergoing oscillation and inertial cavitation disrupt membranes through shear stress or cause increased endocytosis [100], forming a major pathway in the cellular internalization of nanoparticles. Ultrasound-mediated drug delivery and imaging can be combined to effectively target tumors through passive accumulation of AuNPs in tumors and their responsiveness to ultrasound [87,89].

For example, Moon et al. used gold nanocapsules to load hydrophobic or hydrophilic drugs [103]. A phase-change material (PCM) was used as the medium to load the drug so that the drug would not diffuse out of the nanocapsules (produced by galvanic replacement reaction between Ag nanocubes and HAuCl_4_) until the melting point of the PCM was reached. As a result, the release of the drugs could be manipulated by regulating the temperature as long as the drug was miscible in the PCM, which had surfactant-like behavior. The results of the study showed that this combination of PCM and Au nanocapsules could be widely applicable to variety of drugs. HIFU was effective in controlling the temperature of the drug release system and therefore the release profile.

Ultrasound has been used to propel porous gold nanowire motors created by electrodeposition of anodisc alumina membranes coated with poly(sodium 4-styrenesulfonate) (PSS), poly(acrylic acid), or methyl thioglycolate to target tissues [104]. The PSS-coated nanowires had the highest Dox loading capacity, at 17.9 micrograms/membrane, and were able to release 40% of their payload upon exposure to NIR light at 808 nm for 15 min. The movement of the nanowires toward HeLa cells was monitored by microscope as ultrasound waves at 2.01 MHz were applied.

Other nanoconstructs have also been studied. For example, the release of dye from gold nanocages with poly(NIPAAm-co-AAm) thermally-responsive copolymer surface modifications by exposure to HIFU at 1.6 MHz [105]. At a power of 10W, the HIFU irradiation caused most of the dye to be released in 10 minutes, which was much faster than with conventional heating in a 40 °C oil bath. HIFU also caused dye release at a deep penetration depth of 30 mm.

AuNPs have also been used to increase the density of nanomedicine for better ultrasound-mediated drug delivery. A 149-nm adenovirus-Au-PEG (Ad-Au-PEG) construct showed an higher ultrasound-mediated transport resulting from the increased density (3.35 g/mL) of the nanoparticle [106]. As determined by an enzyme-linked immunosorbent assay, Ad-Au-PEG showed good stealthing from antibodies in BALB/c mice that had been implanted with CT26 or HepG2 cancer cells, allowing the development of greater passive accumulation of the particles in vivo. Furthermore, Ad-Au-PEG was found to travel further in a tissue mimicking material flow channel than non-modified Ad and Ad-poly[N-(2-hydroxypropyl)methacrylamide].

### 3.3. Photoacoustic Imaging

PA imaging is an extension of ultrasound, combining standard ultrasound technology with high optical contrast at microscale resolution. Diagnostic PA imaging has gained prominence over the last decade. The primary mechanism of PA imaging is the absorption of electromagnetic energy that translates into the creation of acoustic waves [107]. More specifically, light directed at the tissue is absorbed by photoabsorbers (tuned to a specific wavelength range) that are naturally present in the tissue [108]. This process then causes thermoelastic expansion, generating a wide-band ultrasound wave, which is detected by a transducer and translated into an image representing the location of PA contrast agents [108].

AuNPs are promising contrast agents because they exhibit strong NIF absorption [103,109]. Furthermore, PA spectroscopy can help distinguish contrast agents from other structures of the body [109]. Clinical ultrasound scanners and linear arrays can be used to quickly generate ultrasound and PA images [109] by detecting the ultrasound waves generated by AuNPs through SPR that results from absorption of incident lasers [110]. PA imaging aided by ultrasound can also be used to guide photoabsorber-enhanced photothermal therapy by monitoring temperature and detecting photoabsorbers [111,112].

For clinical applications, the high contrast of PA imaging can reveal early-stage tumors that are undetectable with conventional ultrasound technologies [107]. Furthermore, PA imaging employs electromagnetic energy in the optical and radiofrequency domains because of their non-ionizing properties, high contrast potentials, and adequate penetration depths [107,108]. The imaging depth corresponds to the ultrasonic bandwidth. As the bandwidth increases, for example from 1 to 10 MHz, stronger resolution is attained; however, the ultrasonic penetration depth decreases [107].

Furthermore, PA imaging techniques differ according to the imaging configuration used. In PA depth profiling, the tissue depth structure and properties can be determined from the temporal PA signal. However, PA tomography is more appropriate for imaging more complex structures [107]. This technique is optimal for PA signals measured at different locations around the target material [107].

PA imaging often incorporates contrast agents that improve the quality of the image by accentuating the contrast of the target material [108,113,114]. Contrast agents can be classified as endogenous or exogenous. Much research has focused on endogenous contrasts with chromophores such as hemoglobin and melanin [108]. In one study, PA imaging was used to observe melanoma tumor growth over a 2-week period. Results indicated that greater optical contrast was achieved because there was a higher concentration of melanin in the tumor than in the encompassing tissue [108]. However, exogenous agents produce better accuracy in PA imaging of deeply located tumors [108]. Specifically, exogenous agents can include small-molecule dyes (NIR-absorbing dyes), single-walled carbon nanotubes, AuNPs, or copper nanoparticles [113,114,115]. Each of these contrast agents exhibits different properties and mechanisms that improve the potential of PA imaging. Here, however, the primary focus will be on AuNPs.

AuNPs are of particular interest in PA imaging because they have characteristics related to the SPR effect [108,113]. Because of the SPR effect, AuNPs have absorbance values at a greater magnitude than some of the other exogenous agents [108]. In addition, AuNPs are excitable in the wavelength range of 650–900 nm, which is close to the NIR range, allowing for deeper penetration of light [113]. AuNPs can take a variety of different shapes and sizes, though some forms, such as nanorods, nanoshells, and nanocages, have received more prominence than others in recent research (Table 6) [113,114].

In particular, the standard spherical AuNP has an SPR in the range of visible light. However, as discussed earlier, the SPR must be placed in the infrared range to allow for deeper penetration [113]. The class of nanocages was created to possess wavelength flexibility, meaning that they can be adjusted to reach any wavelength in the infrared range [113]. Nanocages can also be used to enhance PA contrast and control the release of encapsulated materials. For example, a temperature-sensitive PCM has been used to fill the space in the nanocages and encapsulate other chemotherapeutic agents [103]. The material melted upon exposure to heat or HIFU, and the encapsulated dye was released.

In addition to nanocages, nanorods have also been the subject of research in PA imaging. Gold nanorods (AuNRs) with an aspect ratio of 3:1 were used to improve PA imaging contrast [109,115]. Mice dorsal skin flaps that were implanted with human prostate cancer cells (PC3N) were stretched with a window chamber and illuminated with a pulse laser of 680–1000 nm. The skin flaps with the GNRs implanted were scanned using a clinical ultrasound system that provided real-time tracking of contrast agents and volumetric, spectroscopic PA images. A clinical ultrasound scanner and linear array provided 3D images 120 times faster than a single-element mechanical scanning system could. Sites injected with GNRs showed up to an 18-dB increase in PA signal intensity at wavelengths of 825–850 nm, and over 0.8 dB in areas without GNRs. The enhanced system could facilitate PA contrast agent and drug delivery testing while enabling PA imaging and spectroscopy for human cancer research. The ability to dynamically track injection of PA contrast agents could potentially lead to tracking of injections in real time [109]. Nanorods have also been used to modify the surface of lipid-based microbubbles for PA imaging [115].

An ex vivo study used 82-nm Au nanospheres and 25 × 81-nm AuNRs suspended in ultrapure water to test for PA cavitation at 532 nm and 724 nm illuminations, respectively [98]. As laser fluence increased at various pressures, the cavitation probability increased sigmoidally. The concentration of the nanoparticles and the cavitation probability were related logarithmically. The required cavitation threshold fluences (4 mJ/cm^2^ for nanospheres and 2.3 mJ/cm^2^ for nanorods at the mechanical index limit) were below the maximum permissible exposure level in the presence of an applied ultrasound field [9]. Even at relatively low concentrations of AuNPs, cavitation occurred at levels below the maximum permissible exposure for tissue at the mechanical index limit in diagnostic ultrasound.

In another study, alpha(v)beta(3)-gold nanobeacons (AuNBs) were developed for PA tomography of neovasculature [116]. A mouse Matrigel-plug model of angiogenesis was used to demonstrate the efficacy of the GNB. The contrast of GNBs was ten times greater than that of blood in PA tomography. Furthermore, the signal was six times greater than that of hemoglobin using an ultrasound receiver. GNBs allowed for visualization of angiogenic sprouts and bridges, and rhodamine-labeled GNBs specifically targeted immature neovasculature over mature microvasculature.

### 3.4. X-ray Radiation

AuNPs are widely studied and used as a contrast agent for X-ray. Aside from that, AuNPs have been used to enhance the efficacy of radiation therapy owing to its high atomic (Z) number. The proposed mechanism includes photon absorption, photoelectron release and auger electron generation [117]. 

Hainfield et al. [118] intravenously injected 1.9 nm AuNP to tumor bearing animals, and the AuNP enhanced the outcome of 250 kVp X-ray radiation. The one-year long term survival rate of the combination group was as high as 86%, while the long term survival rate of X-ray alone group was only 20%. In a similar study, Jain et al. [119] also used 1.9 mm AuNP but explored the enhancement of megavoltage (MV) X-ray radiation. Their results suggested the enhancement was cell specific. The enhancement was observed in MDA-MB-231 cell, which had the greatest AuNP uptake, but not in L132 or DU145 cells. 

Interestingly, the enhancement of radiation outcome seems to vary according to the radiation source. Kong et al. [120] used 10.8 nm cysteamine- or glucose-coated AuNPs in combination with either X-ray or γ-ray. The results suggested AuNP enhanced cytotoxicity of X-ray but lacked significant effect when combined with γ-rays. However, in a separate study, Zhang et al. [121] used PEG-coated AuNPs of 4.8, 12.1, 27.3 and 46.6 nm in combination with γ-ray. Their results suggested that 12.1 and 27.3 nm AuNPs enhanced the radiation outcome stronger than 4.8 and 46.6 nm AuNPs in vitro and in vivo, and also had higher accumulation in tumor (Table 7).

## 4. Conclusions 

We have reviewed the response of AuNPs to intrinsic stimuli, including pH, MMPs, and GSH, and external stimuli, including laser and ultrasound. The versatility in their response to both intrinsic and extrinsic stimuli makes AuNPs excellent potential tools in cancer imaging and treatment. However, most research ended with showing the efficacy of the AuNPs and little has been reported on the in vivo disposal of the AuNPs after the treatment. Although AuNPs are generally biocompatible, they are not inert and can cause oxidative stress. Thus, the long term toxicity of retained AuNPs must be studied thoroughly in order for a safe clinical application. Similarly, interaction of AuNPs with other blood components, including different hematocytes, serum proteins and other small molecules, requires more study to further understand and evaluate the in vivo behavior and safety of AuNPs. The results from these studies may also in return provide more insight and optimization of the design of AuNPs for cancer theranostics. It will be interesting to see more cancer-related clinical work on AuNPs.

## Figures and Tables

**Table 1:** AuNPs responsive to pH.

Structure and Size at Basic/Physiological pH (7.4)	Structure and Size at Acidic pH	In Vitro Effects	In Vivo Effects	Ref.
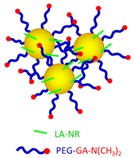 ~80 nm	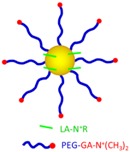 50 nm (pH = 6.8)	Increased cellular uptake at pH 6.8 when compared with pH 7.4	None	[23]
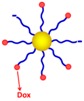 10 nm	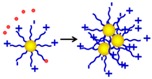 ~250 nm (pH 2.0); ~50 nm (pH 5.5)	Synergistic effect of Dox release and photothermal ablation (660-nm laser)	Significant tumor suppression without noticeable damage to other organs	[24]
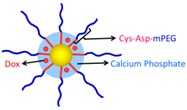 79.8 ± 18.7 nm	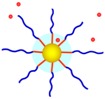 Dissolution of calcium phosphate layer at acidic pH. Size: N/A	Higher cytotoxicity on HeLa cells	None	[27]

**Table 2:** AuNPs responsive to MMP.

Structure and Size before MMP Exposure	Structure after MMP Exposure	Targeting MMP Subclass	In Vitro Effects	In Vivo Effects	Ref.
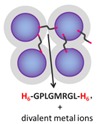 Mean diameter 21.5 nm	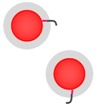	MMP-7	Extinction ratio (E520/E700) as function of MMP concentration	None	[37]
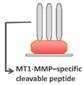 Length 35.2 ± 1.5 nm Width 10.8 ± 0.9 nm	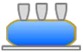	MT1-MMP	LSPR blueshift after MT1-MMP cleavage. λ_max_ and Δλ_max_ depend on MT1-MMP proteolytic activity in cell lysate.	None	[38]
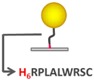 5 nm	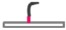	MMP-7	Relative intensity of scanometric image as a function of MMP-7 concentration	None	[39]
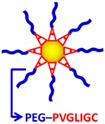 Core 5 nm Whole NP 26 nm	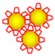 311 nm	MMP-2	Enhanced cellular uptake on MDA-MB-231 cells	None	[40]
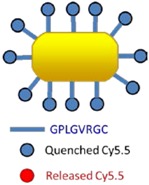 Aspect ratio 2:1	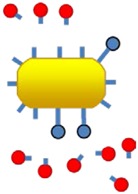	MMP-3, -7, -9, -13	NIRF imaging of MMP by releasing Cy5.5. Cytotoxicity on HeLa cells by photothermal ablation (671 nm CW laser source)	Maximum NIRF intensity 60 min after injection. Temperature exceeded 45 °C after 4 min of irradiation (SCC-7 tumor xenograft)	[41]
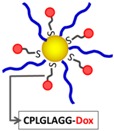 Core 13 nm	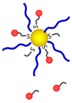	MMP-2 + GSH	Dox release by MMP2 (increased fluorescence), increased cytotoxicity (further intracellular Dox release by GSH) on SCC-7 and HT-29 cells	Increased fluorescence intensity at the tumor site 30 min after injection. Comparable antitumor effect with free Dox, but much lower systemic toxicity and higher animal survival	[42]

GSH, glutathione; LSPR, localized surface plasmon resonance; NIRF, near-infrared fluorescence; CW, continuous wave; Dox, doxorubicin.

**Table 3:** AuNPs responsive to GSH.

Structure and Size before GSH Exposure	Structure and Size after GSH Exposure	In Vitro Effects	In Vivo Effects	Ref.
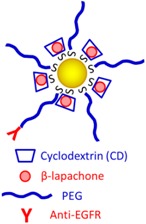 Core 27.0 ± 3 nm–27.6 ± 3 nm	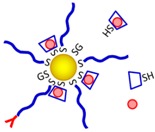	Release of β-lapachone by GSH. Enhanced cellular uptake by anti-EGFR ligand. Enhanced apoptosis than free β-lapachone (A549 cells).	None	[49]
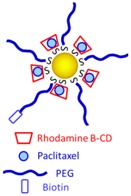 20–40 nm	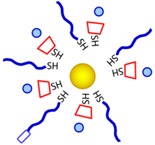	Enhanced cellular uptake on HeLa, A549, and MG63 cell lines. Higher cytotoxicity on HeLa cells as compared with NIH3T3 cells.	None	[50]
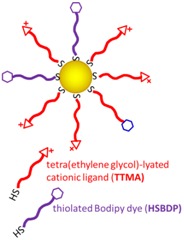 Core 2 nm	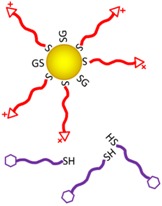	Enhanced cellular uptake by TTMA, and HSBDP release by GSH (HepG2 cells).	None	[51]
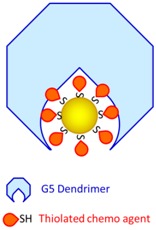 3 nm	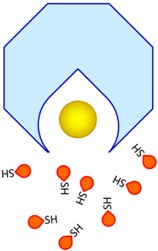	Improved cytotoxicity as compared with free chemo agent (HeLa cells).	None	[52]
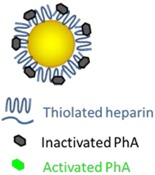 Core 31 nm PhA-heparin/AuNP 40 nm	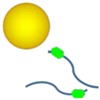	Higher cellular uptake and cytotoxicity of PhA-heparin/AuNP as compared with PhA (A549 cells, 670 nm laser source).	Prolonged circulation, improved tumor specificity, reduced tumor size (15 days) (A549 xenograft on SKH1 nude mice).	[53]
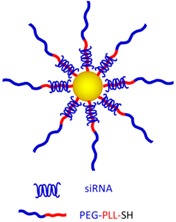 20 nm uPIC-AuNP 38 nm	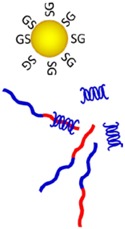	Reduced luciferase activity from gene silencing by siRNA delivery to luciferase-expressing HeLa (HeLa-Luc) cells.	Significant luciferase silencing on HeLa-Luc xenograft.	[54]
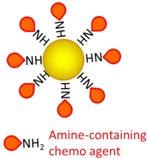 Size varies according to different chemo agent AuNP:MTX 2.6 ± 0.7 nm	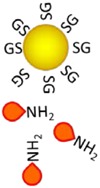	MTX and Au:MTX had similar cytotoxicity on THP-1cell line.	Au:MTX had better leukemia suppression than MTX in a murine xenotransplant model of primary human AML.	[55]
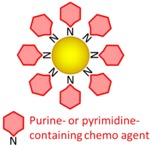 ~20 nm	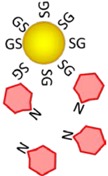	AuNPs containing different chemo agent had drug release and SER intensities after cellular uptake (HeLa cells).	None	[56]
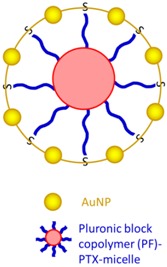 Size of whole NP varied according to formulation	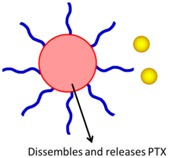	Au-PF-PTX-micelles had higher cytotoxicity on U87 cells pretreated with GSH monoester.	PK and biodistribution studied on BALB/C mice. Au-PF-PTX-micelles preferentially accumulated in spleen and liver.	[57]

uPIC, short interfering RNA-loaded unimer polyion complex; MTX, methotrexate; EGFR, epidermal growth factor receptor; TTMA, tetra(ethylene glycol)-lyated cationic ligand; HSBDP, thiolated Bodipy dye; PhA, pheophorbide A; siRNA, short interfering RNA; SER, surface-enhanced Raman scattering; PF-PTX, paclitaxel loaded Pluronic micelles; and PK, pharmacokinetics.

**Table 4:** Gold nanoparticles (AuNPs) responsive to laser via surface plasmon resonance (SPR).

AuNP Structure and Size	In Vitro Effects	In Vivo Effects	Ref.
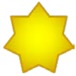 25–150 nm	SPR peaked between 500 and 1000 nm. The dependence of heat efficiency on size and wavelength decreased after cellular uptake (PC3 cells).	The dependence of heat efficiency on size and wavelength decreased only after cellular uptake (intratumoral injection of Au stars, PC3 xenograft).	[69]
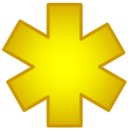 Branched AuNPs Deoxycholate concentration-dependent particle size	cRGD-branched AuNPs decreased cell viability on BxPC3 cells after photothermal ablation (NIR laser source at 808 nm, 1.4 W/cm^2^, for 3 min).	cRGD-branched AuNPs + NIR laser irradiation had the best antitumor effect on BxPC3 xenograft.	[70]
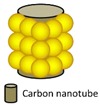 Raw carbon nanotubes 1–2 nm Size of final product varied.	Increased cytotoxicity in combination with NIR irradiation at 808 nm for 15 min (1.6 W, spot size 5 × 20 mm^2^, HeLa cells).	None	[71]
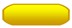 Nanorods 60 × 14.8 ± 6.5 × 2.0 nm 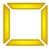 Nanocage edge length 50 ± 7 nm	Compared to nanorod, nanocage had higher light-to-heat transduction efficiencies and higher cellular uptake (HUVEC and DU145 cells).	Compared to nanorod, nanocage had more optimal biodistribution profile over time and higher excretion rate.	[72]
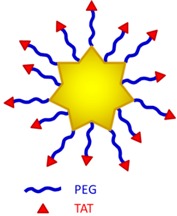	TAT facilitated cellular uptake. Higher photothermolysis efficiency on BT549 breast cancer cells (850 nm pulsed laser source under 0.2 W/cm^2^ irradiation).	None	[73]
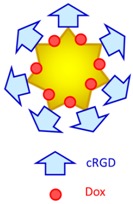 Au nanostar 32.6 nm Au-cRCD-Dox 124.7 nm	cRGD facilitated cellular uptake. Synergistic effect of photothermal therapy and chemotherapy (765 nm high power multimode pump laser, 1.0 W/cm^2^, 10–15 min, MDA-MB-231 and Bel-7402 cells).	Prominent accumulation in tumor and reticuloendothelial system in the liver, and synergistic effect of photothermal therapy and chemotherapy (S180 xenograft).	[74]
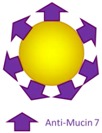 47 nm	Enhanced photothermal therapy outcome on Mucin-7-expressing MBT2, T24, 9202, and 8301 cells at low energy levels (500 exposures, 532 nm laser)	None	[75]
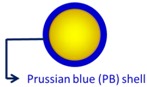 AuNP 9.1 ± 0.64 nm Au@PB NPs 17.8 ± 2.3 nm	Enhanced photothermal cytotoxicity (HeLa cells, NIR 808 nm laser, 1.5W/cm^2^, 10 min). Concentration dependent X-ray, CT, and photoacoustic signals.	Enabled photothermal ablation and simultaneous photoacoustic/CT bimodal imaging (HT-29 xenograft).	[77]
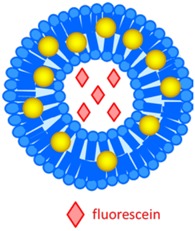 AuNP 3–7 nm AuNP-liposome 100 nm	Laser induced disintegration of liposome and triggered release of fluorescein (fiber-optic guided 65 mW laser, 532 nm).	Higher tumoral retention of fluorescein by liposome as compared to free fluorescein, and fluorescein release triggered by laser (MDA-MB 231 cell xenograft).	[78]
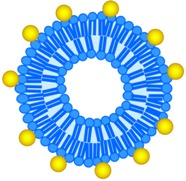 LiposAuNPs 100–120 nm	LiposAuNPs were biocompatible on NIT-3T3 cell line, but exhibited cytotoxicity in combination with laser irradiation (MCF-7 and HT1080 cells).	In situ degradation in hepatocytes and clearance through hepatobiliary and renal routes. Complete tumor ablation using NIR laser (750 nm).	[79]
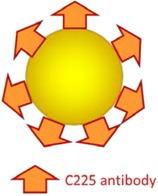 60 nm	Intracellular synergy by (1) nanocluster formation after cellular internalization of AuNPs and TNs; (2) release of the chemo agent upon receiving laser pulse by generation of plasmonic nanobubbles; (3) amplification of X-ray. (HN31 cell lines).	Quadrapeutics system including AuNPs, TNs, laser, and X-ray had the most improved efficacy on fast-growing aggressive HN31 xenograft, as compared with standard chemoradiation.	[80]
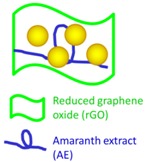 35 nm	Hydrogel shell formation on cells. Enhanced cytotoxicity via combination of photothermal therapy and photodynamic therapy (808 nm, 200 mW/cm^2^, HeLa and Chinese hamster ovary cells).	None	[81]
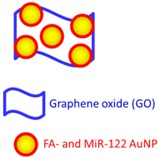 AuNP 5 nm, FA- miR-122-AuNP 20 nm, GO and GGMPN nanocomposites 500 nm	P-gp antibody and FA facilitated cell targeting. Increased apoptosis on drug-resistant HepG2 cells.	Apoptosis induction and tumor growth inhibition on HepG2 xenograft (semiconductor laser light source, 10 min, every 2 days, 10 treatments).	[82]
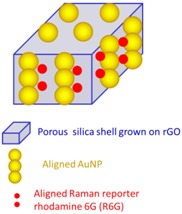 rGO (< 200 nm) Thickness of GO/silica nanosheets 44 nm, AuNP 4 nm	Anti-EGFR SERS probe nanocomposite. Cancer cell tracking by Raman imaging. Enhanced cytotoxicity by synergistic photothermal effect of AuNP and rGO (808 nm laser, 0.5 W/cm^2^, A549 cells).	None	[83]
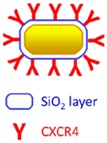 Nanorods length 65.0± 7.5 nm and width 12.0 ± 1.5 nm	Loading of nanorods@ SiO2@CXCR4 into human iPS cells. Reservation of viability of iPS cells and photothermal property of Au nanorods.	Stem cell mediated tumoral delivery, MGC803 xenograft. Prolonged tumoral retention confirmed by photoacoustic and two-photon luminescence imaging. Tumor growth inhibition via photothermal therapy (NIR laser at 808 nm 1.5 W/cm^2^)	[84]
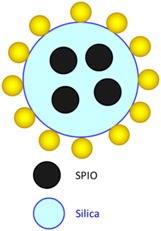 SPIO 10 nm SPIO@AuNPs 82 ± 4 nm	Loading of SPIO@AuNPs into AD-MSCs. Reservation of viability of AD-MSCs and photothermal and magnetic properties of SPIO@AuNPs. Photothermal ablation of HepG2 cells by SPIO@AuNP–loaded AD-MSCs.	Homing of AD-MSCs to liver injuries or HCC confirmed by MR imaging and histologic analysis.	[85]
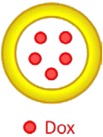 30–50 nm	None	Proved NPs interact with ablative techniques differently. Cellular incorporation of NP was only observed after combination with irreversible electroporation. Structural deformation was only observed in combination with laser-induced thermal therapy (808 nm NIR laser).	[68]

cRGO, cyclic RGD; NIR, near infrared; TAT, TAT peptide; Dox, doxorubicin; PB, Prussian blue; CT, computed tomography; TN, therapeutic nanoparticle; P-gp, P-glycoprotein; FA, folic acid; miRNA, GO, graphene oxide; SERS, surface-enhanced Raman spectroscopy; EGFR, epidermal growth factor receptor; rGO, reduced graphene oxide; iPS, human induced pluripotent stem cells; MR, magnetic resonance; HCC, hepatocellular carcinoma; Ad-MSCs, adipose-derived mesenchymal cells; GGMPN, gold nanoparticles loaded with miR-122; SPIO@AuNPs, superparamagnetic iron oxide-coated gold nanoparticles.

**Table 5:** AuNPs responsive to ultrasound.

Structure and Size of AuNP	In Vitro Effects	In Vivo Effects	Ref.
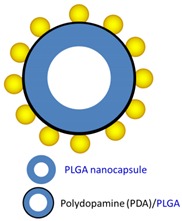 PLGA nanocapsule 690 nm PDA/PLGA 725 nm AuNPs@PDA/PLGA 930 nm	Low cytotoxicity on ECA-109 cells. Increased echogenicity with increased concentration. AuNPs@PDA/PLGA suitable for US imaging.	Highly efficient US guided HIFU ablation ex vivo on degassed bovine liver.	[93]
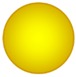 AuNPs 82 ± 6 nm embedded in 7% acrylamide gel phantom	Cavitation activity dependence on HIFU pressure and laser energy was studied.	None	[95]
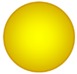 Nanosphere 82 nm 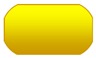 Nanorods 25 nm × 81 nm	PA cavitation vs US pressure and NP concentration was studied. Cavitation threshold fluences decreased with the presence of US. Indicated feasibility of producing PA cavitation in deep tissue within the safe range of US and laser irradiation.	None	[98]
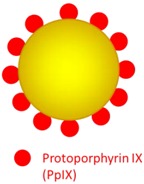 7 nm	None	AuNP-PpIX in combination with US had higher antitumor efficacy.	[102]
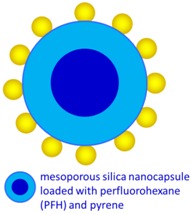 Mesoporous silica nanocapsules 250 nm, with 50 nm shell, AuNP 5–10 nm	Increased cytotoxicity via US induced thermal effect and US induced PFH bubble cavitation and pyrene release (L929 cells). Enhanced grayscale value on US imaging.	Enhanced US imaging of tumor after subcutaneous injection and enhanced HIFU efficacy and accuracy (rabbit VX2 liver tumors).	[99]
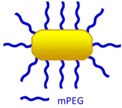 Nanorods 10 × 41 nm, Aspect ratio 4:1 Definity microbubbles	Additive cytotoxicity of US + microbubble and mPEG-Au nanorods + laser.	None	[100]
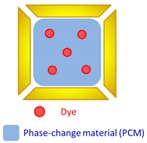 Nanocage outer edge length 60 ± 11 nm, thickness 7.5 ± 2.8 nm	Dye release by HIFU via thermal phase transition of PCM.	None	[103]
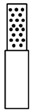 Nanoporous Au wire 250 ± 20 nm	US-powered approaching to HeLa cells. NIR triggered release of Dox.	None	[104]
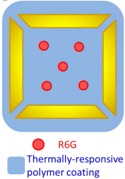 Nanocage edge length 52 nm	Triggered release of R6G by HIFU in gelatin phantom.	Controlled release up to 30 mm covering phantom with chicken breast tissue (ex vivo).	[105]
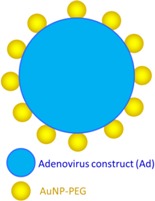 AuNP 6.3 nm AuNP-PEG 15 nm Unmodified Ad 117 nm Ad-AuNP-PEG 149 nm	Increased density of Ad-AuNP-PEG increased response to US induced acoustic cavitation	Higher tumoral accumulation and antitumor effect of Ad-AuNP-PEG + US (HepG2 xenograft).	[106]

PDA, polydopamine; PLGA, poly(lactic-co-glycolic acid); US, ultrasound; HIFU, high intensity focused ultrasound; PA, photoacoustic; PpIX, Protoporphyrin IX; PHF, perfluorohexane; mPEG, methoxy polyethylene glycol; PCM, phase-changing material; NIR, near infrared; Dox, doxorubicin; and Ad, adenovirus.

**Table 6:** Gold nanoparticles (AuNPs) in photoacoustic (PA) imaging.

AuNP	In Vitro Effects	In Vivo Effects	Ref.
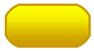 Nanorod 45 nm × 15 nm, Aspect ratio 3:1 Peak absorption near 780 nm	None	Nanorods improved PA imaging contrast (GFP-expressing PC3N tumor, mouse dorsal window chamber).	[109]
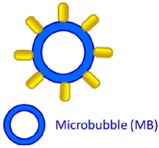 Nanorod 10 × 35 nm	Au nanorod-MBs exhibited increased thermal expansion and over 10-fold greater amplitude of PA signal as compared with Au nanorods. Enhanced PA and pulse inversion images as compared with Au nanorods (gelatin flow phantom).	Enhanced PA and pulse inversion images (murine kidney model).	[115]
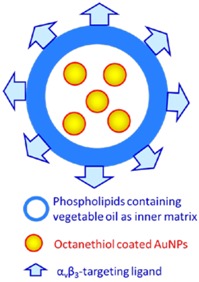 AuNP 3–4 nm Whole NP (α_v_β_3_-Au nanobeacon) 150 nm	Characterization of the size of α_v_β_3_-Au nanobeacon by DLS and AFM.	Sensitive and specific angiogenesis PA imaging (Matrigel-plug mouse model).	[116]

GFP, green fluorescent protein; MB, microbubble; DLS, dynamic light scattering; and AFM, atomic-force microscopy.

**Table 7:** AuNPs sensitizes X-ray radiation.

AuNP	In Vitro Effects	In Vivo Effects	Ref.
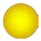 1.9 nm	None	One-year survival rate: 86% with AuNP + X-ray, 20% with X-ray alone, 0% with AuNP alone.	[118]
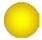 1.9 nm	Greater cellular uptake of AuNP in MDA-MB-231 than in L132 and DU145 cells. Radiation sensitizer enhancement of MV X-ray observed only in MDA-MB-231 cells	None	[119]
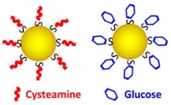 10.8 nm	Cysteamine-AuNP attached to MCF-7 cell membrane. Glucose-AuNP entered MCF-7 cells. Both AuNPs increased cytotoxicity of 200 kVp X-rays but had no significant effect on cytotoxicity of γ-rays.	None	[120]
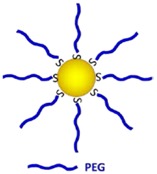 4.8, 12.1, 27.3 and 46.6 nm	12.1 and 27.3 nm AuNPs enhanced the radiation cytotoxicity more than 4.8 and 46.6 nm AuNPs in HeLa cells.	When combined withγ-ray, 12.1 and 27.3 nm AuNPs decreased tumor volume and weight more than 4.8 and 46.6 nm AuNPs U14 tumor model.	[121] *

* The research was in combination with γ-ray; PEG, poly ethylene glycol.

## Abbreviations

The following abbreviations are used in this manuscript:

Ad: adenovirus
Ad-MSCs: adipose-derived mesenchymal cells
AFM: atomic-force microscopy
AuNP: gold nanoparticle
cRGO: cyclic RGD
CT: computed tomography
CW: continuous wave
DLS: dynamic light scattering
Dox: doxorubicin
EGFR: epidermal growth factor receptor
FA: folic acid
GFP: green fluorescent protein
GGMPN: gold nanoparticles loaded with miR-122
GO: graphene oxide
GSH: glutathione
HCC: hepatocellular carcinoma
HIFU: high intensity focused ultrasound
HSBDP: thiolated Bodipy dye
iPS: human induced pluripotent stem cells
LSPR: localized surface plasmon resonance
MB: microbubble
miRNA: microRNA
MMP: matrix metalloproteinases
mPEG: methoxy polyethylene glycol
MR: magnetic resonance
MTX: methotrexate
NIR: near infrared
NIRF: near-infrared fluorescence
PA: photoacoustic
PB: Prussian blue
PCM: phase-changing material
PDA: polydopamine
PF-PTX: paclitaxel loaded Pluronic micelles
P-gp: P-glycoprotein
PhA: pheophorbide A
PHF: perfluorohexane
PK: pharmacokinetics
PLGA: poly(lactic-co-glycolic acid)
PpIX: Protoporphyrin IX
rGO: reduced graphene oxide
SER: surface-enhanced Raman scattering
SERS: surface-enhanced Raman spectroscopy
siRNA: short interfering RNA
SPIO@AuNPs: superparamagnetic iron oxide-coated gold nanoparticles
TAT: TAT peptide
TN: therapeutic nanoparticle
TTMA: tetra(ethylene glycol)-lyated cationic ligand
uPIC: short interfering RNA-loaded unimer polyion complex
US: ultrasound
